# When Surgical Innovation Outpaces Evidence: Does Modern Maximal Resection Require Re-Evaluation of Postoperative Radiotherapy in Glioblastoma?

**DOI:** 10.3390/medsci14030404

**Published:** 2026-07-18

**Authors:** Tomasz Tykocki

**Affiliations:** 1 Department of Paediatric Neurosurgery, Children’s Hospital Named After Prof. Dr Med. Jan Bogdanowicz in Warsaw, 03-924 Warsaw, Poland; ttykocki@gmail.com; 2 Faculty of Medicine, Lazarski Medical University of Warsaw, 02-662 Warsaw, Poland

**Keywords:** glioblastoma, radiotherapy, evidence translation, survival, clinical equipoise

## Abstract

Postoperative radiotherapy (RT) improves survival in glioblastoma, and its role in standard management is not disputed. The randomized trials establishing this benefit, however, were conducted before computed tomography, magnetic resonance imaging (MRI), molecular classification, and temozolomide (TMZ), in heterogeneous populations of “operated malignant glioma” treated with whole-brain or large-field RT versus best supportive care. Their pooled survival benefit (risk ratio 0.81; 95% CI 0.74–0.88) robustly answers the historical question they were designed to address. Since then, advances in surgery, imaging, molecular diagnostics, and systemic therapy have created a modern best-prognosis subgroup—young patients with excellent performance status, MRI-confirmed complete or supramaximal resection of an IDH-wildtype glioblastoma, and median survival approaching or exceeding three years—for whom no clearly defined historical counterpart exists. This perspective provides a structured appraisal of the directness of the landmark randomized evidence using GRADE concepts and translates that appraisal into a graded roadmap for future de-escalation trial designs. Across population, intervention, comparator, and outcomes, the historical trials exhibit substantial indirectness, while the only randomized RT-versus-no-RT evidence from the modern era derives from elderly patients representing the opposite prognostic extreme. This is not an argument against RT. The infiltrative biology of glioblastoma, predominantly in-field recurrence, and radioresistant stem-cell populations strongly support continued benefit. Rather, the unresolved question concerns the magnitude of benefit after maximal contemporary therapy and whether selected de-escalation strategies merit prospective evaluation. Our thesis is one of collective scientific equipoise regarding an unresolved evidence question rather than individual clinician equipoise or refutation of current standard care.

## 1. Introduction

For two decades, glioblastoma management has followed a single template: maximal safe resection, followed by involved-field radiotherapy at 60 Gy with concurrent and adjuvant temozolomide [1,2]. This is among the best-validated regimens in neuro-oncology, and nothing in this paper questions that radiotherapy prolongs survival in glioblastoma. The narrower question we raise concerns external validity: the foundational claim that postoperative irradiation extends survival derives from randomized trials assembled before any of the tools that define modern practice existed—before routine computed tomography, before magnetic resonance imaging (MRI), before the molecular taxonomy of the 2021 World Health Organization (WHO) classification, and before temozolomide [3]. The pooled estimate that anchors guideline recommendations comes from six such trials, conducted largely in the 1970s and 1980s [4]. These six randomized comparisons constitute the complete body of randomized evidence comparing postoperative radiotherapy with no radiotherapy or contemporary non-radiotherapy control available in the Cancer Care Ontario systematic review by Laperriere et al., which remains the only comprehensive synthesis of this historical evidence base. No additional randomized RT-versus-no-RT trials in newly diagnosed glioblastoma have subsequently altered this evidence base.

**Figure 1:**
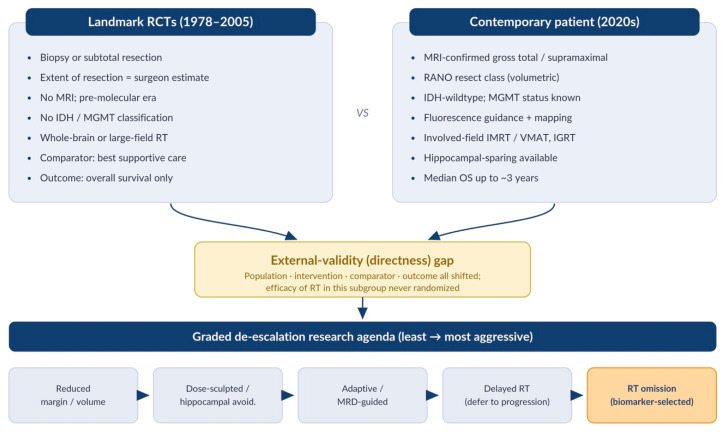
The external-validity gap in postoperative radiotherapy for glioblastoma. The landmark randomized trials enrolled a patient and treatment profile (**left**) that modern surgery, imaging and molecular classification have since replaced (**right**). Because population, intervention, comparator and outcome have all shifted, the efficacy of radiotherapy in the contemporary maximally resected subgroup has never been directly randomized—the directness gap (**centre**)—a question that a graded sequence of de-escalation designs, from reduced-margin radiotherapy to biomarker-selected omission, could address. IGRT, image-guided radiotherapy; IMRT, intensity-modulated radiotherapy; MRD, minimal residual disease; OS, overall survival; RANO, Response Assessment in Neuro-Oncology; VMAT, volumetric-modulated arc therapy.

Over the same interval, neurosurgery has been transformed. Intraoperative MRI, fluorescence guidance, functional mapping and volumetric response-assessment frameworks have redefined what “maximal resection” means and which patients achieve it, producing a contemporary best-prognosis subgroup—young [5,6], high-performance-status patients with MRI-confirmed complete or supramaximal resection of an IDH-wildtype tumour—that did not exist as an identifiable, reproducible category when the radiotherapy evidence was generated. The question is whether the landmark evidence remains directly applicable to that subgroup, and I argue that this is a genuine question of evidence applicability rather than a demonstrated error in practice. The distinction matters: the argument is about what has and has not been tested rather than whether the standard of care is correct. Figure 1 summarizes the divergence between the trial-era population and the contemporary patient, and the research agenda it motivates. The novelty of this perspective is therefore not the observation that the landmark trials predated MRI or molecular classification, but its systematic appraisal of how each component of the historical evidence differs from the contemporary clinical question, and the translation of that appraisal into a practical hierarchy of future randomized trial designs.

## 2. The Randomized Basis for Postoperative Radiotherapy Is Historical and Indirect

### 2.1. Internal Validity Is Not External Validity

A landmark randomized trial can retain excellent internal validity—an unbiased estimate of the effect it measured in the patients it enrolled—while progressively losing external validity as the disease definition, imaging, surgical technique and adjuvant therapy around it evolve [7]. Internal validity is fixed at the moment of randomization; external validity is contingent on how closely the trial population and conditions still resemble the patient in front of the clinician. The question raised here is therefore not whether the foundational radiotherapy trials were sound—as they were—but whether their results remain directly transportable to a glioblastoma population that modern surgery, imaging and molecular pathology have redefined. Directness, in the evidence-based-medicine sense, is precisely this property: the GRADE framework recognizes indirectness when the population, the intervention, the comparator or the outcome of the available evidence differ from those of the question being asked—and the evidence in Table 1 differs on all four [8].

### 2.2. Indirectness, and Transportability, Are Matters of Degree

External validity is not binary, and the argument here is deliberately not that the historical estimate is invalid [7]. A relative effect may well transport across populations, although this assumption depends on the absence of important effect modification [15,16]. Questioning external validity does not imply that the historical treatment effect is necessarily smaller or larger in contemporary patients; rather, it acknowledges that this assumption has never been directly tested in the modern MRI-defined and molecularly characterized population.

The biological case presented in Section 6 suggests that radiotherapy continues to confer benefit after maximal resection. The question is quantitative rather than qualitative: not whether radiotherapy remains beneficial, but whether the magnitude of its benefit is preserved after profound changes in baseline prognosis, extent of resection, molecular classification, and competing therapies [17]. Because absolute treatment benefit depends on the underlying event rate in the untreated population, substantial improvements in prognosis may alter the absolute benefit–harm balance even if the relative treatment effect remains broadly constant [15]. Here, the author raises the directness question in that spirit: not to overturn the estimate, but to ask whether it can be carried, unmodified, to a population the trials never sampled.

Several contemporary observational studies are broadly consistent with continued benefit from postoperative radiotherapy after maximal resection. However, because treatment allocation is influenced by patient selection, molecular profile, performance status, and clinician judgement, these studies cannot isolate the independent contribution of radiotherapy or determine whether the magnitude of benefit remains unchanged in the contemporary best-prognosis subgroup.

### 2.3. Effect Modification Versus Prognosis

This formulation hinges on a distinction worth making explicit, because a reviewer may reasonably object that a better-prognosis patient does not necessarily experience a different treatment effect. The transportability concern rests on the assumption that extent of resection, molecular status or improved baseline prognosis might modify the relative or absolute effect of radiotherapy—that is, that they act as effect modifiers rather than as mere prognostic factors [16]. A prognostic factor shifts the baseline risk in both treatment arms without necessarily altering the relative treatment effect, whereas an effect modifier changes the magnitude of the treatment effect itself [18]. These concepts are frequently conflated. Whether extent of resection and molecular status function solely as prognostic factors or also modify the effect of postoperative radiotherapy remains unknown because this question has never been tested in the contemporary maximally resected subgroup. That uncertainty is not a weakness of the present argument but its central premise: it defines an evidence gap that only a contemporary randomized trial can resolve.

The recommendation to irradiate after surgery rests on a pooled analysis of six randomized trials of RT versus no RT, which reported a survival benefit favouring radiotherapy (risk ratio 0.81; 95% CI 0.74–0.88) [4]. That estimate is real and, for its era, robust. Its constituent trials share a common structure that becomes problematic only when the result is transported to the present: extent of resection was a surgeon’s impression rather than an imaging measurement; irradiation was frequently whole-brain or large-field rather than the involved-field technique used today; and the comparator was best supportive care or first-generation nitrosourea chemotherapy [9,10,11,12,13]. Table 1 appraises each trial against the question relevant to a contemporary maximally resected patient, identifying the axes on which the extrapolation becomes indirect.

### 2.4. Indirectness Across the Clinical Question

The trials in Table 1 are indirect with respect to the modern question on every axis at once: on population (no image-verified resection, no IDH or MGMT stratification, and—in the Scandinavian and Sandberg–Wollheim trials [13]—inclusion of grade III astrocytomas that under WHO 2021 would no longer be diagnosed as glioblastoma [3]), on intervention (whole-brain or large-field RT without a temozolomide backbone), on comparator (best supportive care or nitrosourea chemotherapy, not modern temozolomide-based management with RT deferred) [9,10,11], and on outcome (overall survival alone, with the late-neurocognitive and quality-of-life endpoints that motivate de-escalation never captured) [14,19,20]. Viewed through a GRADE lens, evidence of this configuration would be expected to suffer marked indirectness with respect to the modern target population [8,21]. The author makes this observation illustratively, to characterize the applicability problem, rather than as a formal certainty rating: a formal GRADE assessment requires systematic-review methodology against a pre-specified question and is not what is offered here.

### 2.5. The Keime–Guibert Paradox

The single randomization that is recognizably modern—conducted in the CT/MRI era, using involved-field RT in a clean RT-versus-no-RT design—was performed in elderly, frail, frequently biopsy-only patients, comparing RT against best supportive care [14]. The field’s only contemporary direct evidence on radiotherapy versus no radiotherapy thus sits precisely in the population least able to inform the good-prognosis, maximally resected decision—indirectness not at the margin of the evidence base but at its centre.

## 3. Modern Maximal Resection Has Redefined the Patient Population

**Figure 2:**
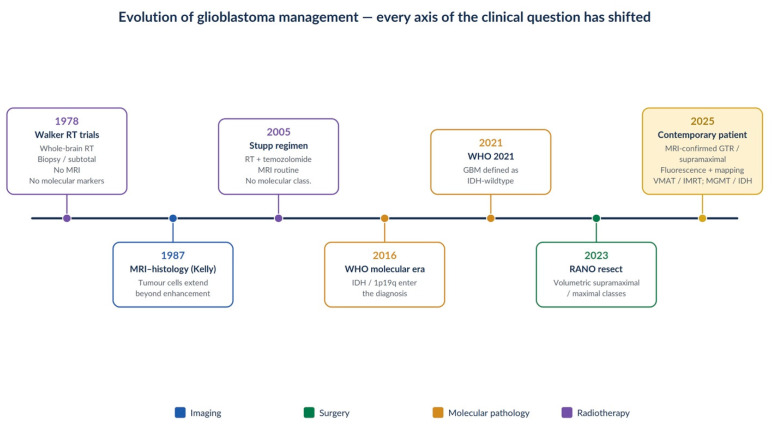
Evolution of glioblastoma management, 1978–2025. Each milestone changed a different axis of the clinical question—radiotherapy technique and surgical extent, the availability of MRI, and the molecular definition of the disease—so that the contemporary patient (highlighted) differs from the trial-era patient in every dimension, not surgery alone. GBM, glioblastoma; GTR, gross total resection; IDH, isocitrate dehydrogenase; IMRT, intensity-modulated radiotherapy; MGMT, O6-methylguanine-DNA methyltransferase; RANO, Response Assessment in Neuro-Oncology; RT, radiotherapy; VMAT, volumetric-modulated arc therapy; WHO, World Health Organization.

The most consequential change since the foundational trials has been in surgery. Extent of resection, once based largely on the surgeon’s intraoperative assessment, is now a volumetric, MRI-verified, and prognostically validated variable [22]. The RANO (Response Assessment in Neuro-Oncology) resect classification, derived from more than 1000 patients with IDH-wildtype glioblastoma treated according to the Stupp regimen, stratifies resection into supramaximal (class 1), maximal contrast-enhancing (class 2), submaximal (class 3), and biopsy (class 4), and remains independently associated with overall survival after adjustment for established molecular and clinical prognostic factors [23]. Within that cohort, supramaximal resection was associated with longer median overall survival than maximal contrast-enhancing resection (24 vs. 19 months). An independent reconstruction of individual patient data further emphasized this prognostic separation, reporting a median overall survival of 35.6 months for supramaximal resection compared with 13.9 months for all lesser resection categories (hazard ratio 0.28) [24]. Importantly, these advances have not simply improved prognosis; they have created a reproducibly identifiable subgroup that could not have been prospectively defined or stratified in the landmark randomized radiotherapy trials. Surgery, however, is only one of several dimensions that have evolved. As illustrated in Figure 2, imaging, molecular classification, and radiotherapy techniques have also changed fundamentally, so that the contemporary target population differs substantially from that represented in the historical trials. Consequently, the question is not whether those trials were internally valid, but whether their treatment-effect estimates can be directly transported to today’s MRI-defined, molecularly characterized population.

These figures describe a population with no historically well-defined or reproducibly identifiable analogue: young, well-resected patients have always existed, but they could not be reliably identified, volumetrically verified or molecularly defined when the radiotherapy evidence was generated. A patient with median survival exceeding two-and-a-half years bears little resemblance to the “operated malignant glioma” patient of Walker or Andersen, whose median survival was measured in weeks [9,11]. The molecular dimension compounds the discontinuity: because the 2021 WHO classification requires IDH-wildtype status for a glioblastoma diagnosis, several historical trials enrolling grade III–IV astrocytomas included tumours that would today be diagnosed as IDH-mutant astrocytoma and managed differently [12,13]. Evidence base and target population have drifted apart on the patient axis as much as on the technical one. It is worth emphasizing that although the RANO resect categories have been validated prognostically [24], they were devised to standardize the reporting of surgical extent and to stratify trials, not to guide adjuvant radiotherapy decisions; they are invoked here to characterize the modern population, not to assign treatment.

A clarification on what defines the relevant subgroup is therefore warranted, and two distinct populations should be kept separate. The external-validity argument itself applies to all MRI-confirmed maximal resections—any patient in whom complete or supramaximal resection has been volumetrically verified—because the question of whether historical evidence transports does not depend on molecular status; it follows simply from the mismatch between the trial-era population and the modern, image-verified one. The population in which radiotherapy de-escalation might eventually be ethically and feasibly *tested*, by contrast, is narrower, and depends on the following: the conjunction of low residual disease (MRI-confirmed complete or supramaximal resection, classified by RANO resect category), favourable tumour biology (IDH-wildtype with MGMT promoter methylation) [25], and favourable host factors (younger age, high performance status) [26]. Extent of resection alone is necessary but not sufficient to delineate that trial-eligible group; MGMT methylation and host factors enter not because the appraisal requires them, but because they identify the patients in whom a de-escalation question could responsibly be asked [27]. Conflating the two populations—the broad one to which the directness concern applies and the narrow one in which a trial could be run—would weaken both claims.

## 4. Modern Radiotherapy Is Not Historical Radiotherapy

Symmetry requires acknowledging that radiotherapy has changed as much as surgery. The foundational trials delivered whole-brain or large-field irradiation; contemporary practice uses involved-field, intensity-modulated or volumetric-arc therapy with daily image guidance, reduced clinical-target-volume margins, and, where critical structures are at risk, dose-sculpting techniques such as hippocampal avoidance, which preserved cognition in a randomized trial in brain metastases [19]. This modernization offers two ways for the present argument, and honesty demands stating both.

On one hand, it weakens the late-toxicity rationale for de-escalation. If modern conformal RT spares far more normal brain than the whole-brain RT of the 1970s, the cognitive cost that de-escalation seeks to avoid is smaller than historical experience implies, and the case for withholding RT is correspondingly weaker [19,28]. On the other hand, technique modernization does not remotely address the core of the directness problem, which concerns efficacy in a subgroup rather than mode of delivery: no degree of conformality answers whether RT adds a survival benefit after MRI-confirmed maximal resection, because that comparison has never been made. Reduced-margin conformal RT even raises its own untested question—whether tighter fields adequately cover infiltrative disease beyond the enhancing margin [29]. Modern RT thus changes the toxicity side of the ledger and the geometry of delivery, but leaves the efficacy gap in the maximally resected subgroup exactly where it was.

## 5. What Modern Data Do—And Do Not—Tell Us About Radiotherapy After Maximal Resection

### 5.1. The Pivotal Trial Isolates Chemotherapy, Not Radiotherapy

It is tempting to argue from the Stupp trial that well-resected patients benefit most from the standard regimen. That trial, however, randomized patients to radiotherapy alone versus identical radiotherapy plus temozolomide; every patient was irradiated [2,16]. Any subgroup advantage by extent of resection therefore quantifies the benefit of adding temozolomide on a universal radiotherapy background—it cannot isolate the marginal contribution of radiotherapy within the maximally resected subgroup. A trial that isolates that contribution has not been performed.

### 5.2. Observational Data Cannot Substitute

Several contemporary observational cohorts and registry analyses support the continued use of postoperative radiotherapy after maximal resection. However, treatment allocation in these studies is strongly influenced by patient selection, molecular profile, performance status, and clinician judgement, making confounding by indication unavoidable. Consequently, observational studies remain hypothesis-generating and cannot isolate the independent causal contribution of radiotherapy or determine whether its incremental benefit differs within the contemporary best-prognosis subgroup. Only randomization can address that question directly [30].

### 5.3. The Neurocognitive Cost Is Plausible but, in the Modern Era, Contested

The de-escalation rationale assumes that long-surviving, well-resected patients live long enough to accrue late radiation effects. The contemporary evidence is genuinely mixed: some prospective series find that modern partial-brain irradiation affects neurocognition less than once assumed, and the elderly de-escalation trials that measured cognition and quality of life found them comparable between arms [14,20]. Combined with the conformality gains noted above [19], this makes the late-harm argument real but unproven for modern involved-field RT in good-prognosis patients—a reason for equipoise, not a demonstration of harm.

## 6. Why the Evidence Gap May Not Change the Conclusion

Several independent biological and clinical observations strongly support the continued benefit of postoperative radiotherapy even after maximal resection, and intellectual honesty requires giving them full weight. First, glioblastoma is diffusely infiltrative, and its cellular extent exceeds any imaging boundary: stereotactic biopsy studies correlating histology with imaging showed isolated tumour-cell infiltration extending at least as far as the T2 signal, well beyond the contrast-enhancing rim that defines a “gross total” resection [31]. Removing all of the enhancing tumour therefore leaves precisely the microscopic peritumoral disease that radiotherapy is designed to sterilize. Second, the pattern of failure confirms this: in glioblastoma treated with radiotherapy and temozolomide, recurrence is predominantly central and in-field—within the treated volume—rather than distant, with roughly three-quarters of failures arising inside the radiation field [32]. Third, the cells most likely to repopulate the tumour are the least radiosensitive—glioma stem-like cells preferentially activate the DNA-damage checkpoint and survive irradiation in increased proportion, an intrinsic radioresistance that surgery cannot address [33].

Taken together, these observations provide a compelling biological rationale for postoperative radiotherapy even after MRI-confirmed maximal resection, because surgery cannot eradicate infiltrative microscopic disease, in-field recurrence remains the dominant pattern of failure, and glioma stem-like cells exhibit intrinsic radioresistance [31,32,33]. The argument of this paper is therefore not that radiotherapy is dispensable—the available biological and clinical evidence suggests otherwise—but that the magnitude of its incremental benefit in the contemporary maximally resected subgroup has never been isolated by randomized evidence [4,7]. Whether that benefit remains quantitatively unchanged in a population with markedly improved baseline prognosis is therefore unknown and represents the central evidence gap identified in this appraisal

## 7. Why the Question Has Remained Unanswered

If the evidence gap is genuine, why has no trial addressed it in two decades? The reasons are themselves informative. Contemporary international guidelines uniformly recommend postoperative chemoradiotherapy for eligible patients with newly diagnosed glioblastoma, making individual clinician equipoise uncommon despite continued collective scientific uncertainty regarding the precise magnitude of radiotherapy benefit in carefully selected contemporary subgroups [1,34]. The expectation of predominantly local recurrence, grounded in the infiltrative biology of glioblastoma, further reduces willingness to defer or omit radiotherapy in patients perceived to have the greatest potential to benefit. Moreover, the target population—patients with MRI-confirmed complete or supramaximal resection, favourable molecular characteristics, younger age, and preserved performance status—represents only a minority of an already uncommon disease, implying that any adequately powered trial would require extensive international collaboration [35]. Finally, the ethical challenges inherent in randomizing patients away from an established standard of care remain substantial, particularly for complete omission of radiotherapy. These barriers explain why the evidence gap has persisted, but they do not eliminate it; rather, they argue for carefully designed, biomarker-selected, multicenter trials instead of indefinite reliance on historical extrapolation.

## 8. Reframing the Question: A Spectrum of Trial Designs

Framing the gap as a single provocative comparison—standard chemoradiotherapy versus temozolomide alone—understates the available options and invites the objection that such a trial is unethical. The gap, in fact, admits a spectrum of de-escalation designs of increasing aggressiveness. These are, from least to most aggressive, as follows: reduced-target-volume or reduced-margin radiotherapy, testing whether infiltrative-disease margins inherited from the pre-MRI era can be safely tightened after maximal resection [29,31]; dose-sculpted or hippocampal-avoidance radiotherapy, testing whether late toxicity can be lowered without sacrificing control; adaptive or response/minimal-residual-disease–guided radiotherapy, modulating dose or volume to imaging or molecular signals [36]; delayed radiotherapy, deferring treatment to documented progression under close surveillance; and, at the most aggressive end and with the steepest ethical and equipoise barriers, radiotherapy omission with temozolomide alone, defensible only within a tightly defined biomarker-selected stratum (for example MGMT-methylated, supramaximally resected tumours) under a non-inferiority design [25].

The less aggressive designs are testable now with broad acceptability; the omission design is the one reviewers will rightly scrutinize, and it belongs at the end of a research programme, not its opening move. Table 2 sets out these candidate designs against the considerations that determine whether each is worth pursuing—ethical acceptability, feasibility, novelty and potential impact—and makes explicit that the proposal is a graded agenda, with outright omission positioned as its hardest and last step rather than its premise. A search of ClinicalTrials.gov identified no ongoing randomized trial evaluating planned omission or deferral of postoperative radiotherapy in younger patients with newly diagnosed maximally resected glioblastoma.

Assuming a conventional non-inferiority design with overall survival as the primary endpoint, acceptable non-inferiority margins would need to be conservative, and the resulting sample size would likely be substantial, necessitating international multicentre collaboration. Whichever design is chosen, overall survival should remain the primary endpoint, with neurocognition, quality of life, progression-free survival, recurrence pattern, time to radiotherapy, and salvageability included as co-primary or key secondary endpoints. Stratification by RANO resect class and MGMT status would align the analysis with the contemporary prognostic architecture [23,24]. The qualitative ratings for ethical acceptability, feasibility, novelty and potential impact represent the author’s structured judgement based on four considerations: consistency with current international guidelines, anticipated clinician acceptance, expected logistical complexity of conducting an adequately powered multicentre trial, and the degree to which each design departs from current standard practice”.

## 9. Limitations and Generalizability

This analysis is a critical appraisal of applicability, not new outcome data. It demonstrates that the historical randomized evidence is indirect with respect to the modern maximally resected patient and that no contemporary randomization addresses that subgroup; it does not, and cannot, show that radiotherapy should be withheld—and the biology of Section 6 suggests it should not be, outside a trial. The limitation is intrinsic to the genre: an external-validity argument identifies what has not been tested, leaving the empirical question open by construction.

The phenomenon is not unique to radiotherapy in glioblastoma. The external validity of a randomized trial has long been recognized as a question separate from its internal validity, and one that is routinely under-reported and under-examined [7]. Wherever a technological or methodological advance materially changes the population, the intervention, or the measurable outcomes after a landmark trial was completed, the transportability of that trial’s effect to the contemporary patient becomes a legitimate empirical question rather than a settled fact [15]. Surgical and procedural fields are especially exposed, because the index intervention itself evolves: time-threshold rules, volume thresholds and adjuvant-therapy norms set in earlier eras may or may not transfer to populations defined by modern imaging, technique and molecular classification. Radiotherapy after maximal glioblastoma resection is one well-characterized instance, and the directness framework used here—specifying a modern target population and scoring each landmark trial against it [8]—offers a transferable template for appraising aging randomized evidence in any rapidly evolving surgical discipline.

## 10. Conclusions

Postoperative radiotherapy improves survival in glioblastoma; that is not in question. What is in question is whether the randomized evidence establishing it remains directly applicable to a population that surgery, imaging and molecular classification have created since those trials were run—a population that achieves MRI-confirmed maximal resection, survives far longer than any historical cohort, and was never represented in the trials that govern its treatment. The landmark randomized evidence contains important elements of indirectness when applied to this contemporary subgroup, and its one modern RT-versus-no-RT instance comes from the opposite prognostic group. The biology of infiltration, in-field recurrence and stem-cell radioresistance suggests that radiotherapy still helps; the magnitude of that help in the modern subgroup, and its balance against late cost, has simply never been measured. Recognizing an evidence gap does not invalidate current practice; it defines the next question that prospective trials should answer.

## Figures and Tables

**Table 1:** Directness appraisal of the randomized RT-versus-no-RT evidence against the MRI-confirmed gross-total-resection question.

Trial (Era)	Population and How Resection Was Judged	Radiotherapy + Systemic Background	Comparator Arm	Outcomes Captured	Where Indirectness Enters for the GTR Question
Walker/BTSG 69-01, 1978; J Neurosurg 49:333 [9]	Histologically confirmed anaplastic glioma; surgeon-impression of operability; pre-CT/pre-MRI; no molecular classification	Whole-brain RT 50–60 Gy via bilateral opposing ports; ±BCNU; no temozolomide	Best conventional care—no RT, no chemotherapy	Overall survival only (≈14 vs. ≈35 wk)	Indirect on all four PICO axes: no image-verified GTR, no molecular stratum, whole-brain field, comparator is no treatment, cognition/QoL unmeasured
Walker/BTSG 72-01, 1980; N Engl J Med 303:1323 [10]	Malignant glioma after surgery; surgeon-assessed extent; pre-MRI/pre-molecular	Large-field RT; randomized against nitrosourea regimens	Nitrosourea-only (no-RT) arms	Overall survival only	Same four-axis indirectness; “no-RT” contrast is 1970s chemotherapy, not modern TMZ ± observation
Andersen, 1978; Acta Radiol Oncol 17:475 [11]	Glioblastoma; randomized postoperative series; no imaging of extent of resection	Postoperative RT, era technique	No postoperative RT	Overall survival	No GTR isolation; pre-molecular; OS-only; small historical series
Kristiansen/SGSG, 1981; Cancer 47:649 [12]	Operated astrocytoma grade III–IV—includes tumours now classified as IDH-mutant astrocytoma, not GBM	RT ± bleomycin	No RT	Overall survival	Population partly reclassified out of GBM; no MRI/molecular; OS-only
Sandberg-Wollheim, 1991; Cancer 68:22 [13]	Astrocytoma grade ¾	PCV ± RT	PCV without RT	Overall survival	RT effect entangled with PCV; no GTR stratification; pre-molecular
Keime-Guibert, 2007; N Engl J Med 356:1527 [14]	Elderly ≥ 70; biopsy or resection; CT/MRI era but extent of resection not the stratifier; frail	Involved-field (focal) RT; best-supportive-care background	Best supportive care	OS (≈6.7 vs. 3.9 mo); QoL/cognition measured	The only contemporary RT-vs-no-RT RCT—but in the opposite prognostic population; deepens rather than closes the gap

**Reference question:** newly diagnosed IDH-wildtype glioblastoma; MRI-confirmed complete or supramaximal resection; younger age, high KPS, MGMT-methylated; involved-field: 60 Gy versus identical management minus RT; outcomes: overall survival and neurocognition/quality of life. Pooled estimate of rows 1–5: RR 0.81 (95% CI 0.74–0.88), favouring postoperative RT. BTSG, Brain Tumor Study Group; GTR, gross total resection; KPS, Karnofsky performance status; PCV, procarbazine–lomustine–vincristine; QoL, quality of life; RR, risk ratio; SGSG, Scandinavian Glioblastoma Study Group; TMZ, temozolomide.

**Table 2:** Candidate de-escalation trial designs, ordered from least to most aggressive.

Candidate Design	Ethical Acceptability	Feasibility	Novelty	Potential Impact	Existing Supporting Evidence
Reduced target volume/margin	High	High	Moderate	Moderate	*Limited retrospective*
Dose-sculpted/hippocampal-avoidance RT	High	High	Moderate	Moderate	*Strong in brain metastases; none in GBM*
Adaptive/MRD-guided RT	Moderate	Moderate	High	High	*Early feasibility only*
Delayed RT (defer to progression)	Moderate	Moderate	High	High	*None*
RT omission (biomarker-selected)	Low	Low	Very high	Very high	*None*

Ratings are qualitative and indicative; the final column summarizes the current evidence base supporting each design, underscoring that the proposal is grounded in existing knowledge and that radiotherapy omission—least acceptable, least feasible, and unsupported by direct evidence—is positioned as the endpoint of a programme, not its opening move. MRD, minimal residual disease; RT, radiotherapy.

## Data Availability

The original contributions presented in this study are included in the article. Further inquiries can be directed to the corresponding author.

## References

[1] Stupp R., Hegi M.E., Mason W.P., van den Bent M.J., Taphoorn M.J.B., Janzer R.C., Ludwin S.K., Allgeier A., Fisher B., Belanger K. (2009). Effects of radiotherapy with concomitant and adjuvant temozolomide versus radiotherapy alone on survival in glioblastoma in a randomised phase III study: 5-year analysis of the EORTC-NCIC trial. Lancet Oncol..

[2] Stupp R., Mason W.P., van den Bent M.J., Weller M., Fisher B., Taphoorn M.J.B., Belanger K., Brandes A.A., Marosi C., Bogdahn U. (2005). Radiotherapy plus concomitant and adjuvant temozolomide for glioblastoma. N. Engl. J. Med..

[3] Louis D.N., Perry A., Wesseling P., Brat D.J., Cree I.A., Figarella-Branger D., Hawkins C., Ng H.K., Pfister S.M., Reifenberger G. (2021). The 2021 WHO Classification of Tumors of the Central Nervous System: A summary. Neuro-Oncology.

[4] Laperriere N., Zuraw L., Cairncross G. (2002). Cancer Care Ontario Practice Guidelines Initiative Neuro-Oncology Disease Site Group. Radiotherapy for newly diagnosed malignant glioma in adults: A systematic review. Radiother. Oncol..

[5] Tykocki T., Rakasz Ł. (2026). Connectome-guided glioma resection: A systematic review of white matter tract preservation and postoperative neurocognition. Neurosurg. Rev..

[6] Stummer W., Pichlmeier U., Meinel T., Wiestler O.D., Zanella F., Reulen H.-J. (2006). ALA-Glioma Study Group. Fluorescence-guided surgery with 5-aminolevulinic acid for resection of malignant glioma: A randomised controlled multicentre phase III trial. Lancet Oncol..

[7] Rothwell P.M. (2005). External validity of randomised controlled trials: “To whom do the results of this trial apply?”. Lancet.

[8] Guyatt G., Oxman A., Kunz R., Woodcock J., Brozek J., Helfand M., Alonso-Coello P. (2011). GRADE guidelines: 8. Rating the quality of evidence—Indirectness. J. Clin. Epidemiol..

[9] Walker M., Alexander E., Hunt W. (1978). Evaluation of BCNU and/or radiotherapy in the treatment of anaplastic gliomas: A cooperative clinical trial. J. Neurosurg..

[10] Walker M., Green S., Byar D. (1980). Randomized comparisons of radiotherapy and nitrosoureas for the treatment of malignant glioma after surgery. N. Engl. J. Med..

[11] Andersen A. (1978). Postoperative irradiation of glioblastomas: Results in a randomized series. Acta Radiol. Oncol. Radiat. Phys. Biol..

[12] Kristiansen K., Hagen S., Kollevold T. (1981). Combined modality therapy of operated astrocytomas grade III and IV. A prospective multicenter trial of the Scandinavian Glioblastoma Study Group. Cancer.

[13] Sandberg-Wollheim M., Malmström P., Strömblad L. (1991). A randomized study of chemotherapy with procarbazine, vincristine, and lomustine with and without radiation therapy for astrocytoma grades 3 and/or 4. Cancer.

[14] Keime-Guibert F., Chinot O., Taillandier L. (2007). Radiotherapy for glioblastoma in the elderly. N. Engl. J. Med..

[15] Dahabreh I., Hernán M. (2019). Extending inferences from a randomized trial to a target population. Eur. J. Epidemiol..

[16] VanderWeele T. (2009). On the distinction between interaction and effect modification. Epidemiology.

[17] Kent D.M., Rothwell P.M., Ioannidis J.P.A., Altman D.G., Hayward R.A. (2010). Assessing and reporting heterogeneity in treatment effects in clinical trials: A proposal. Trials.

[18] Sun X., Ioannidis J.P.A., Agoritsas T., Alba A.C., Guyatt G. (2014). How to use a subgroup analysis: Users’ guide to the medical literature. JAMA.

[19] Brown P., Gondi V., Pugh S. (2020). Hippocampal avoidance during whole-brain radiotherapy plus memantine for patients with brain metastases: Phase III trial NRG Oncology CC001. J. Clin. Oncol..

[20] Perry J., Laperriere N., O’Callaghan C. (2017). Short-course radiation plus temozolomide in elderly patients with glioblastoma. N. Engl. J. Med..

[21] Schünemann H.J., Oxman A.D., Brozek J., Glasziou P., Jaeschke R., Vist G.E., Williams J.W., Kunz R., Craig J., Montori V.M. (2008). Grading quality of evidence and strength of recommendations for diagnostic tests and strategies. BMJ.

[22] Lacroix M., Abi-Said D., Fourney D.R., Gokaslan Z.L., Shi W., DeMonte F., Lang F.F., McCutcheon I.E., Hassenbusch S.J., Holland E. (2001). A multivariate analysis of 416 patients with glioblastoma multiforme: Prognosis, extent of resection, and survival. J. Neurosurg..

[23] Karschnia P., Young J., Dono A. (2023). Prognostic validation of a new classification system for extent of resection in glioblastoma: A report of the RANO resect group. Neuro-Oncology.

[24] Wach J., Vychopen M., Güresir E. (2025). Prognostic revalidation of RANO categories for extent of resection in glioblastoma: A reconstruction of individual patient data. J. Neurooncol..

[25] Hegi M.E., Diserens A.-C., Gorlia T., Hamou M.-F., de Tribolet N., Weller M., Kros J.M., Hainfellner J.A., Mason W., Mariani L. (2005). MGMT gene silencing and benefit from temozolomide in glioblastoma. N. Engl. J. Med..

[26] Weller M., van den Bent M., Tonn J.C., Stupp R., Preusser M., Cohen-Jonathan-Moyal E., Henriksson R., Le Rhun E., Balana C., Chinot O. (2017). European Association for Neuro-Oncology (EANO) guideline on the diagnosis and treatment of adult astrocytic and oligodendroglial gliomas. Lancet Oncol..

[27] Malmström A., Grønberg B.H., Marosi C., Stupp R., Frappaz D., Schultz H., Abacioglu U., Tavelin B., Lhermitte B., Hegi M.E. (2012). Temozolomide versus standard 6-week radiotherapy versus hypofractionated radiotherapy in patients older than 60 years with glioblastoma: The Nordic randomised, phase 3 trial. Lancet Oncol..

[28] Hingorani M., Colley W.P., Dixit S., Beavis A.M. (2012). Hypofractionated radiotherapy for glioblastoma: Strategy for poor-risk patients or hope for the future? *Br*. J. Radiol..

[29] Niyazi M., Andratschke N., Bendszus M., Chalmers A.J., Erridge S.C., Galldiks N., Lagerwaard F.J., Navarria P., Munck Af Rosenschöld P., Ricardi U. (2023). ESTRO-EANO guideline on target delineation and radiotherapy details for glioblastoma. Radiother. Oncol..

[30] Hernán M.A., Robins J.M. (2016). Using Big Data to Emulate a Target Trial When a Randomized Trial Is Not Available. Am. J. Epidemiol..

[31] Kelly P., Daumas-Duport C., Kispert D., Kall B., Scheithauer B., Illig J. (1987). Imaging-based stereotaxic serial biopsies in untreated intracranial glial neoplasms. J. Neurosurg..

[32] Brandes A., Tosoni A., Franceschi E. (2009). Recurrence pattern after temozolomide concomitant with and adjuvant to radiotherapy in newly diagnosed patients with glioblastoma: Correlation with MGMT promoter methylation status. J. Clin. Oncol..

[33] Bao S., Wu Q., McLendon R. (2006). Glioma stem cells promote radioresistance by preferential activation of the DNA damage response. Nature.

[34] Cabrera A.R., Kirkpatrick J.P., Fiveash J.B., Shih H.A., Koay E.J., Lutz S., Petit J., Chao S.T., Brown P.D., Vogelbaum M. (2016). Radiation therapy for glioblastoma: Executive summary of an American Society for Radiation Oncology Evidence-Based Clinical Practice Guideline. Pract. Radiat. Oncol..

[35] Watanabe S., Nonaka T., Yamada M., Maeda M., Sugii N., Arakawa Y., Hashimoto K., Ishikawa E. (2026). Efficacy Endpoint Standardization in Adult Primary CNS Tumor Trials: Integrating Regulatory Science and Clinical Perspectives in the RANO 2.0 Era. Cancers.

[36] Tsien C., Cao Y., Chenevert T. (2014). Clinical applications for diffusion magnetic resonance imaging in radiotherapy. Semin. Radiat. Oncol..

